# 4-{[8-(4-Acetyl­oxybenzo­yl)-2,7-dimeth­oxy­naphthalen-1-yl]carbon­yl}phenyl acetate

**DOI:** 10.1107/S160053681203228X

**Published:** 2012-07-21

**Authors:** Kosuke Sasagawa, Daichi Hijikata, Taro Kusakabe, Akiko Okamoto, Noriyuki Yonezawa

**Affiliations:** a Department of Organic and Polymer Materials Chemistry, Tokyo University of Agriculture & Technology, Koganei, Tokyo 184-8588, Japan

## Abstract

In the mol­ecule of the title compound, C_30_H_24_O_8_, the two 4-acet­oxy­benzoyl groups at the 1- and 8-positions of the naphthalene ring system are aligned almost anti­parallel, and the two benzene rings make a dihedral angle of 54.21 (9)°. The dihedral angles between the benzene rings and the naphthalene ring system are 63.63 (8) and 78.54 (8)°.

## Related literature

For formation reactions of aroylated naphthalene compounds *via* electrophilic aromatic substitution of naphthalene derivatives, see: Okamoto *et al.* (2009 ▶, 2011 ▶). For the structures of closely related compounds, see: Hijikata *et al.* (2010 ▶); Muto, Kato *et al.* (2010 ▶); Sasagawa, Hijikata *et al.* (2011 ▶); Sasagawa, Muto *et al.* (2011 ▶); Muto, Sasagawa *et al.* (2012 ▶).
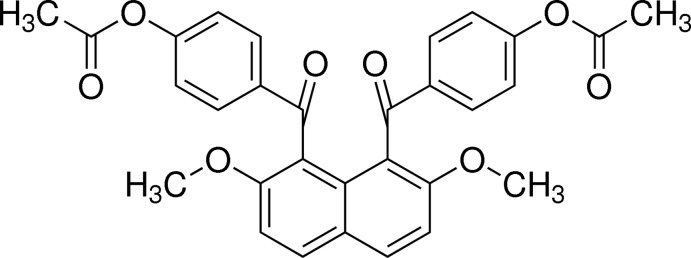


## Experimental

### 

#### Crystal data

C_30_H_24_O_8_
*M* *_r_* = 512.49
Monoclinic, 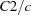

*a* = 44.115 (6) Å
*b* = 7.9710 (9) Å
*c* = 15.035 (4) Å
β = 99.439 (16)°
*V* = 5215.2 (15) Å^3^
*Z* = 8
Cu *K*α radiation
μ = 0.79 mm^−1^
*T* = 193 K
0.60 × 0.20 × 0.05 mm

#### Data collection

Rigaku R-AXIS RAPID diffractometer
Absorption correction: numerical (*NUMABS*; Higashi, 1999 ▶) *T*_min_ = 0.649, *T*_max_ = 0.962
44265 measured reflections
4760 independent reflections
3547 reflections with *I* > 2σ(*I*)
*R*_int_ = 0.024

#### Refinement

*R*[*F* ^2^ > 2σ(*F* ^2^)] = 0.041
*wR*(*F* ^2^) = 0.127
*S* = 1.11
4760 reflections
348 parameters
H-atom parameters constrained
Δρ_max_ = 0.21 e Å^−3^
Δρ_min_ = −0.23 e Å^−3^

### 

Data collection: *PROCESS-AUTO* (Rigaku, 1998 ▶); cell refinement: *PROCESS-AUTO*; data reduction: *CrystalStructure* (Rigaku, 2010 ▶); program(s) used to solve structure: *SIR2004* (Burla *et al.*, 2005 ▶); program(s) used to refine structure: *SHELXL97* (Sheldrick, 2008 ▶); molecular graphics: *ORTEPIII* (Burnett & Johnson, 1996 ▶); software used to prepare material for publication: *SHELXL97*.

## Supplementary Material

Crystal structure: contains datablock(s) I, global. DOI: 10.1107/S160053681203228X/pk2431sup1.cif

Structure factors: contains datablock(s) I. DOI: 10.1107/S160053681203228X/pk2431Isup2.hkl

Supplementary material file. DOI: 10.1107/S160053681203228X/pk2431Isup3.cml

Additional supplementary materials:  crystallographic information; 3D view; checkCIF report

## Figures and Tables

**Table 1:** Hydrogen-bond geometry (Å, °)

*D*—H⋯*A*	*D*—H	H⋯*A*	*D*⋯*A*	*D*—H⋯*A*
C11—H11*C*⋯O4^i^	0.98	2.36	3.320 (3)	166
C12—H12*A*⋯O3^ii^	0.98	2.53	3.380 (3)	145
C3—H3⋯O7^iii^	0.95	2.47	3.369 (3)	158
C21—H21⋯O8^iv^	0.95	2.53	3.364 (3)	146

Symmetry codes: (i) 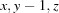; (ii) 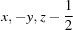; (iii) 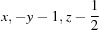; (iv) 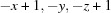.

**Fig. 1.**
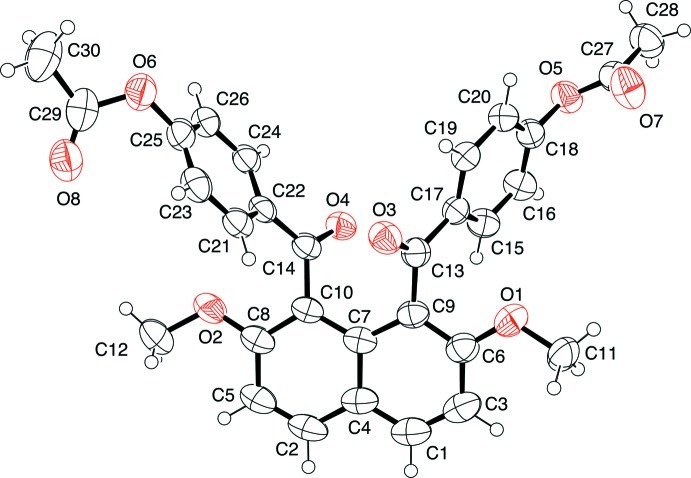
Molecular structure with displacement ellipsoids drawn at the 50% probability level.

**Fig. 2.**
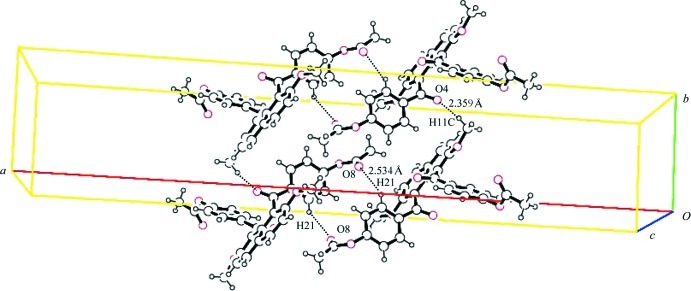
Intermolecular C—H···O interactions between H11C and O4 [symmetry equivalent x,1 + y,z] and between H21 and O8 [symmetry equivalent -x,1 - y,-z].

## supplementary crystallographic information

### Comment

In the course of our study on selective electrophilic aromatic aroylation of the naphthalene ring core, 1,8-diaroylnaphthalene compounds have proved to be formed regioselectively by the aid of a suitable acidic mediator (Okamoto & Yonezawa, 2009, Okamoto *et al.*, 2011). Recently, we have reported the X-ray crystal structures of 1,8-diaroylated 2,7-dimethoxynaphthalene derivatives such as [2,7-dimethoxy-8-(4-methylbenzoyl)-1-naphthyl](4-methylphenyl)methanone [1,8-bis(4-methylbenzoyl)-2,7-dimethoxynaphthalene] (Muto *et al.*, 2010), [2,7-dimethoxy-8-(2,4,6-trimethylbenzoyl)naphthalen-1-yl](2,4,6-trimethylphenyl)methanone [1,8-bis(2,4,6-trimethylbenzoyl)-2,7-dimethoxynaphthalene] (Muto *et al.*, 2012), {8-[4-(bromomethyl)benzoyl]-2,7-dimethoxynaphthalen-1-yl}[4-(bromomethyl)phenyl]methanone [1,8-bis(4-bromomethylbenzoyl)-2,7-dimethoxynaphthalene] (Sasagawa, Hijikata *et al.*, 2011), and {8-[4-(butoxy)benzoyl]-2,7-dimethoxynaphthalen-1-yl}[4-(butoxy)phenyl]methanone [1,8-bis(4-butoxylbenzoyl)-2,7-dimethoxynaphthalene] (Sasagawa, Muto *et al.*, 2011). The aroyl groups in these compounds are almost perpendicularly attached to the naphthalene rings and oriented in opposite directions (*anti*-orientation). Moreover, we have also shown that the aroyl groups of 2,7-dimethoxy-1,8-bis(4-phenoxybenzoyl)naphthalene (Hijikata *et al.*, 2010) are oriented in the same direction (*syn*-orientation) in the crystal. As part of our ongoing studies on the molecular structures of these kinds of homologous molecules, the X-ray crystal structure of the title compound, 1,8-diaroylated naphthalene bearing acetoxy groups, is discussed in this article.

The molecular structure of the title compound is displayed in Fig 1. Two 4-acetoxybenzoyl groups are twisted away from the attached naphthalene ring and are situated in *anti* orientation. The dihedral angle between the best planes of the two phenyl rings is 54.21 (9)°. The two dihedral angles between the best planes of the 4-acetoxyphenyl rings and the naphthalene ring are 63.63 (8) and 78.54 (8)°, respectively.

The dihedral angles between the naphthalene ring system and the bridging ketonic carbonyl C—C(═O)—C planes [58.30 (9) and 54.11 (9)°] are larger than those between the phenyl rings and the bridging carbonyl planes [10.65 (10) and 28.80 (10)°]. Besides, the dihedral angles between the phenyl rings and the bridging acetoxy C—C(═O)—O planes [57.29 (10) and 60.32 (13)°] are similar to those between the naphthalene ring system and the bridging ketonic carbonyl C—C(═O)—O planes.

In the molecular packing, four C—H···O interactions are observed, *i.e.*, two types of C—H···O interactions between the oxygen atoms of the ketonic carbonyl groups and the hydrogen atoms of the methoxy groups [C11—H11C···O4 = 2.36 Å, C12—H12A···O3 = 2.53 Å], C—H···O interaction between carbonyl oxygen atom of the acetoxy groups and hydrogen atom of the napthalene ring [C3—H3···O7 = 2.47 Å], and C—H···O interaction between carbonyl oxygen atom of the acetoxy group and hydrogen atom of the benzene ring [C21—H21···O8 = 2.53 Å]. The C—H···O interactions between the methoxy group and the ketonic carbonyl group and between the acetoxy group and the benzene ring effectively contribute to stabilization of the molecular packing (Fig. 2).

### Experimental

The title compound was prepared by an esterification reaction of 1,8-bis(4-hydroxybenzoyl)-2,7-dimethoxynaphthalene (1.0 mmol, 428.5 mg), which was obtained *via* S_N_Ar reaction of 1,8-bis(4-fluorobenzoyl)-2,7-dimethoxynaphthalene with sodium hydroxide, with acetic anhydride (63.0 mmol, 6.43 g) in the presence of concentrated sulfuric acid (1 drop). After the reaction mixture was stirred at room temperature for 1 h, it was poured into water (30 ml). The aqueous layer was extracted with CHCl_3_ (15 ml × 3).The combined extracts were washed with aqueous NaHCO_3_ followed by washing with brine. The organic layers thus obtained were dried over anhydrous MgSO_4_. The solvent was removed under reduced pressure to give a cake. The crude product was purified by recrystallization from methanol (isolated yield 56%). The isolated product was crystallized from methanol to give single-crystals.

^1^H NMR δ (300 MHz, CDCl_3_); 2.30 (6*H*, s), 3.70 (6*H*, s), 7.07 (4*H*, d, *J* = 8.4 Hz), 7.20 (2*H*, d, *J*= 8.7 Hz), 7.69 (4*H*, d, *J* = 8.1 Hz), 7.95 (2*H*, d, *J* = 9.0 Hz) p.p.m. ^13^C NMR δ(75 MHz, CDCl_3_); 21.20, 56.31, 110.03, 120.88, 121.03, 125.36, 120.72, 130.56, 132.18,136.12, 153.94, 156.27, 168.72, 195.69 p.p.m. IR(KBr); 1760(C═O, ester), 1662(C═O, ketone), 1609, 1511, 1461(Ar, napthalene) cm^-1^. (m/*z*): [*M* + H]+ Calcd for C_30_H_25_O_8_, 513.1549; found, 513.1545. M.p. = 434.4 - 436.9 K

### Refinement

All H atoms were found in a difference map and were subsequently refined as riding atoms, with C—H = 0.95 (aromatic) and 0.98 (methyl) Å, and with *U*_ĩso_(H)= 1.2 *U*_eq_(C). Displacement parameters of atoms C6 and O1 were restrained using the *SHELXL97* commands *DELU* and *SIMU*.

### Crystal data

**Table:**

C_30_H_24_O_8_	*F*(000) = 2144
*M**_r_* = 512.49	*D*_x_ = 1.305 Mg m^−^^3^
Monoclinic, *C*2/*c*	Melting point = 436.9–434.4 K
Hall symbol: -C 2yc	Cu *K*α radiation, λ = 1.54178 Å
*a* = 44.115 (6) Å	Cell parameters from 2415 reflections
*b* = 7.9710 (9) Å	θ = 3.0–66.9°
*c* = 15.035 (4) Å	µ = 0.79 mm^−^^1^
β = 99.439 (16)°	*T* = 193 K
*V* = 5215.2 (15) Å^3^	Platelet, colorless
*Z* = 8	0.60 × 0.20 × 0.05 mm

### Data collection

**Table:**

Rigaku R-AXIS RAPID diffractometer	4760 independent reflections
Radiation source: rotating anode	3547 reflections with *I* > 2σ(*I*)
Graphite monochromator	*R*_int_ = 0.024
Detector resolution: 10.000 pixels mm^-1^	θ_max_ = 68.1°, θ_min_ = 4.1°
ω scans	*h* = −52→51
Absorption correction: numerical (*NUMABS*; Higashi, 1999)	*k* = −9→9
*T*_min_ = 0.649, *T*_max_ = 0.962	*l* = −18→18
44265 measured reflections	

### Refinement

**Table:**

Refinement on *F*^2^	Secondary atom site location: difference Fourier map
Least-squares matrix: full	Hydrogen site location: inferred from neighbouring sites
*R*[*F*^2^ > 2σ(*F*^2^)] = 0.041	H-atom parameters constrained
*wR*(*F*^2^) = 0.127	*w* = 1/[σ^2^(*F*_o_^2^) + (0.0509*P*)^2^ + 3.8146*P*] where *P* = (*F*_o_^2^ + 2*F*_c_^2^)/3
*S* = 1.11	(Δ/σ)_max_ = 0.003
4760 reflections	Δρ_max_ = 0.21 e Å^−^^3^
348 parameters	Δρ_min_ = −0.23 e Å^−^^3^
0 restraints	Extinction correction: *SHELXL97* (Sheldrick, 2008), Fc^*^=kFc[1+0.001xFc^2^λ^3^/sin(2θ)]^-1/4^
Primary atom site location: structure-invariant direct methods	Extinction coefficient: 0.00093 (6)

### Special details

**Table:**

Geometry. All e.s.d.'s (except the e.s.d. in the dihedral angle between two l.s. planes) are estimated using the full covariance matrix. The cell e.s.d.'s are taken into account individually in the estimation of e.s.d.'s in distances, angles and torsion angles; correlations between e.s.d.'s in cell parameters are only used when they are defined by crystal symmetry. An approximate (isotropic) treatment of cell e.s.d.'s is used for estimating e.s.d.'s involving l.s. planes.
Refinement. Refinement of *F*^2^ against ALL reflections. The weighted *R*-factor *wR* and goodness of fit *S* are based on *F*^2^, conventional *R*-factors *R* are based on *F*, with *F* set to zero for negative *F*^2^. The threshold expression of *F*^2^ > σ(*F*^2^) is used only for calculating *R*-factors(gt) *etc*. and is not relevant to the choice of reflections for refinement. *R*-factors based on *F*^2^ are statistically about twice as large as those based on *F*, and *R*- factors based on ALL data will be even larger.

### Fractional atomic coordinates and isotropic or equivalent isotropic displacement parameters (Å^2^)

**Table:**

	*x*	*y*	*z*	*U*_iso_*/*U*_eq_	
O1	0.67019 (4)	−0.50504 (18)	0.69478 (11)	0.0666 (4)	
O2	0.57758 (4)	0.0498 (2)	0.38430 (9)	0.0684 (4)	
O3	0.60948 (3)	−0.21880 (19)	0.69000 (9)	0.0577 (4)	
O4	0.63836 (3)	0.07349 (17)	0.58412 (9)	0.0530 (3)	
O5	0.73159 (3)	0.02768 (17)	0.94108 (10)	0.0600 (4)	
O6	0.50983 (3)	0.3687 (2)	0.63806 (10)	0.0713 (5)	
O7	0.72389 (4)	−0.1669 (2)	1.04398 (11)	0.0749 (5)	
O8	0.48041 (4)	0.3266 (2)	0.50231 (13)	0.0765 (5)	
C1	0.65066 (5)	−0.5185 (3)	0.44900 (17)	0.0674 (6)	
H1	0.6537	−0.5870	0.3995	0.081*	
C2	0.61596 (5)	−0.3398 (3)	0.34495 (14)	0.0663 (6)	
H2	0.6187	−0.4115	0.2964	0.080*	
C3	0.66475 (5)	−0.5624 (3)	0.53323 (18)	0.0662 (6)	
H3	0.6775	−0.6588	0.5423	0.079*	
C4	0.63189 (5)	−0.3764 (3)	0.43240 (15)	0.0577 (5)	
C5	0.59705 (5)	−0.2061 (3)	0.32843 (14)	0.0647 (6)	
H5	0.5858	−0.1876	0.2697	0.078*	
C6	0.65994 (5)	−0.4615 (3)	0.60696 (15)	0.0556 (5)	
C7	0.62858 (4)	−0.2672 (3)	0.50575 (12)	0.0493 (5)	
C8	0.59414 (5)	−0.0946 (3)	0.39916 (13)	0.0547 (5)	
C9	0.64307 (4)	−0.3145 (2)	0.59433 (13)	0.0488 (5)	
C10	0.61025 (4)	−0.1195 (2)	0.48571 (12)	0.0474 (4)	
C11	0.68463 (6)	−0.6645 (3)	0.71381 (19)	0.0754 (7)	
H11A	0.6885	−0.6830	0.7791	0.090*	
H11B	0.7041	−0.6667	0.6907	0.090*	
H11C	0.6711	−0.7532	0.6847	0.090*	
C12	0.55560 (6)	0.0640 (4)	0.30369 (15)	0.0797 (7)	
H12A	0.5663	0.0748	0.2518	0.096*	
H12B	0.5428	0.1633	0.3074	0.096*	
H12C	0.5426	−0.0364	0.2964	0.096*	
C13	0.63614 (4)	−0.2268 (2)	0.67754 (12)	0.0477 (4)	
C14	0.61272 (4)	0.0255 (2)	0.54981 (12)	0.0454 (4)	
C15	0.69160 (5)	−0.1424 (3)	0.72838 (14)	0.0544 (5)	
H15	0.6964	−0.1745	0.6714	0.065*	
C16	0.71455 (5)	−0.0820 (3)	0.79466 (14)	0.0573 (5)	
H16	0.7350	−0.0725	0.7833	0.069*	
C17	0.66159 (4)	−0.1561 (2)	0.74484 (12)	0.0451 (4)	
C18	0.70746 (5)	−0.0359 (2)	0.87711 (13)	0.0511 (5)	
C19	0.65498 (5)	−0.1042 (2)	0.82785 (13)	0.0495 (5)	
H19	0.6344	−0.1107	0.8391	0.059*	
C20	0.67777 (5)	−0.0431 (3)	0.89443 (14)	0.0537 (5)	
H20	0.6730	−0.0070	0.9508	0.064*	
C21	0.55710 (4)	0.0299 (3)	0.56628 (12)	0.0517 (5)	
H21	0.5555	−0.0848	0.5490	0.062*	
C22	0.58486 (4)	0.1138 (2)	0.56932 (11)	0.0453 (4)	
C23	0.53169 (5)	0.1133 (3)	0.58840 (13)	0.0574 (5)	
H23	0.5128	0.0560	0.5879	0.069*	
C24	0.58705 (5)	0.2828 (3)	0.59350 (12)	0.0501 (5)	
H24	0.6061	0.3399	0.5961	0.060*	
C25	0.53453 (5)	0.2814 (3)	0.61117 (13)	0.0568 (5)	
C26	0.56166 (5)	0.3679 (3)	0.61376 (13)	0.0557 (5)	
H26	0.5630	0.4835	0.6291	0.067*	
C27	0.73804 (5)	−0.0490 (3)	1.02320 (15)	0.0575 (5)	
C28	0.76456 (5)	0.0327 (3)	1.08052 (16)	0.0713 (6)	
H28A	0.7587	0.1460	1.0965	0.086*	
H28B	0.7819	0.0392	1.0473	0.086*	
H28C	0.7706	−0.0333	1.1356	0.086*	
C29	0.48345 (5)	0.3866 (3)	0.57648 (19)	0.0667 (6)	
C30	0.46120 (6)	0.4943 (4)	0.6140 (2)	0.0937 (9)	
H30A	0.4635	0.4768	0.6793	0.112*	
H30B	0.4402	0.4649	0.5859	0.112*	
H30C	0.4652	0.6123	0.6017	0.112*	

### Atomic displacement parameters (Å^2^)

**Table:**

	*U*^11^	*U*^22^	*U*^33^	*U*^12^	*U*^13^	*U*^23^
O1	0.0778 (10)	0.0442 (8)	0.0766 (11)	0.0067 (7)	0.0087 (8)	−0.0012 (7)
O2	0.0918 (11)	0.0719 (10)	0.0371 (8)	0.0067 (9)	−0.0022 (7)	−0.0024 (7)
O3	0.0519 (8)	0.0720 (10)	0.0501 (8)	−0.0014 (7)	0.0106 (6)	−0.0031 (7)
O4	0.0542 (8)	0.0500 (8)	0.0525 (8)	−0.0057 (6)	0.0020 (6)	−0.0068 (6)
O5	0.0662 (9)	0.0515 (8)	0.0579 (9)	−0.0124 (7)	−0.0027 (7)	−0.0004 (7)
O6	0.0650 (9)	0.0921 (12)	0.0572 (9)	0.0241 (8)	0.0109 (7)	0.0053 (8)
O7	0.0694 (10)	0.0795 (11)	0.0707 (10)	−0.0193 (9)	−0.0040 (8)	0.0163 (9)
O8	0.0638 (10)	0.0704 (11)	0.0880 (13)	−0.0015 (8)	−0.0094 (9)	0.0064 (9)
C1	0.0698 (14)	0.0650 (14)	0.0716 (16)	−0.0075 (12)	0.0239 (12)	−0.0233 (12)
C2	0.0724 (14)	0.0812 (16)	0.0477 (12)	−0.0163 (13)	0.0172 (11)	−0.0253 (11)
C3	0.0625 (13)	0.0501 (12)	0.0892 (18)	−0.0022 (10)	0.0220 (12)	−0.0146 (12)
C4	0.0594 (12)	0.0574 (12)	0.0603 (13)	−0.0092 (10)	0.0213 (10)	−0.0180 (10)
C5	0.0711 (14)	0.0807 (16)	0.0423 (11)	−0.0103 (13)	0.0090 (10)	−0.0139 (11)
C6	0.0560 (11)	0.0474 (11)	0.0632 (13)	−0.0043 (9)	0.0096 (10)	−0.0061 (10)
C7	0.0523 (10)	0.0518 (11)	0.0456 (11)	−0.0108 (9)	0.0132 (8)	−0.0090 (9)
C8	0.0615 (12)	0.0643 (13)	0.0393 (10)	−0.0108 (10)	0.0109 (9)	−0.0074 (9)
C9	0.0491 (10)	0.0440 (10)	0.0543 (11)	−0.0061 (8)	0.0117 (9)	−0.0067 (8)
C10	0.0545 (11)	0.0512 (11)	0.0377 (10)	−0.0081 (9)	0.0107 (8)	−0.0060 (8)
C11	0.0712 (15)	0.0450 (12)	0.0991 (19)	0.0075 (11)	−0.0183 (13)	−0.0118 (12)
C12	0.0871 (17)	0.100 (2)	0.0459 (13)	0.0052 (15)	−0.0069 (12)	−0.0032 (12)
C13	0.0539 (11)	0.0444 (10)	0.0453 (10)	0.0006 (8)	0.0090 (8)	0.0037 (8)
C14	0.0552 (11)	0.0450 (10)	0.0353 (9)	−0.0050 (8)	0.0057 (8)	0.0006 (8)
C15	0.0583 (11)	0.0536 (12)	0.0524 (12)	−0.0067 (9)	0.0125 (9)	−0.0041 (9)
C16	0.0560 (11)	0.0577 (12)	0.0589 (13)	−0.0107 (10)	0.0114 (10)	−0.0020 (10)
C17	0.0515 (10)	0.0363 (9)	0.0467 (10)	0.0013 (8)	0.0055 (8)	0.0026 (8)
C18	0.0577 (11)	0.0397 (10)	0.0526 (11)	−0.0037 (9)	−0.0012 (9)	0.0000 (8)
C19	0.0527 (11)	0.0452 (10)	0.0502 (11)	0.0048 (8)	0.0070 (9)	−0.0006 (8)
C20	0.0616 (12)	0.0497 (11)	0.0488 (11)	0.0037 (9)	0.0058 (9)	−0.0044 (9)
C21	0.0589 (12)	0.0564 (12)	0.0379 (10)	−0.0024 (9)	0.0025 (8)	0.0008 (9)
C22	0.0524 (10)	0.0516 (11)	0.0300 (9)	−0.0001 (8)	0.0006 (7)	−0.0009 (8)
C23	0.0538 (11)	0.0736 (15)	0.0438 (11)	−0.0015 (10)	0.0049 (9)	0.0069 (10)
C24	0.0559 (11)	0.0530 (11)	0.0388 (10)	−0.0006 (9)	0.0000 (8)	0.0015 (8)
C25	0.0597 (12)	0.0694 (14)	0.0404 (11)	0.0152 (11)	0.0055 (9)	0.0053 (10)
C26	0.0642 (13)	0.0551 (12)	0.0449 (11)	0.0084 (10)	0.0004 (9)	0.0005 (9)
C27	0.0588 (12)	0.0546 (12)	0.0567 (13)	−0.0021 (10)	0.0023 (10)	−0.0029 (10)
C28	0.0694 (14)	0.0717 (15)	0.0671 (15)	−0.0109 (12)	−0.0056 (11)	−0.0074 (12)
C29	0.0533 (12)	0.0697 (15)	0.0782 (17)	0.0033 (11)	0.0141 (12)	0.0230 (13)
C30	0.0704 (16)	0.107 (2)	0.110 (2)	0.0262 (16)	0.0331 (15)	0.0328 (18)

### Geometric parameters (Å, º)

**Table:**

O1—C6	1.369 (3)	C12—H12B	0.9800
O1—C11	1.430 (3)	C12—H12C	0.9800
O2—C8	1.362 (3)	C13—C17	1.493 (3)
O2—C12	1.427 (3)	C14—C22	1.487 (3)
O3—C13	1.223 (2)	C15—C16	1.385 (3)
O4—C14	1.225 (2)	C15—C17	1.390 (3)
O5—C27	1.365 (3)	C15—H15	0.9500
O5—C18	1.407 (2)	C16—C18	1.377 (3)
O6—C29	1.371 (3)	C16—H16	0.9500
O6—C25	1.407 (2)	C17—C19	1.390 (3)
O7—C27	1.198 (3)	C18—C20	1.378 (3)
O8—C29	1.200 (3)	C19—C20	1.385 (3)
C1—C3	1.362 (3)	C19—H19	0.9500
C1—C4	1.402 (3)	C20—H20	0.9500
C1—H1	0.9500	C21—C23	1.390 (3)
C2—C5	1.351 (3)	C21—C22	1.389 (3)
C2—C4	1.416 (3)	C21—H21	0.9500
C2—H2	0.9500	C22—C24	1.395 (3)
C3—C6	1.413 (3)	C23—C25	1.384 (3)
C3—H3	0.9500	C23—H23	0.9500
C4—C7	1.431 (3)	C24—C26	1.385 (3)
C5—C8	1.408 (3)	C24—H24	0.9500
C5—H5	0.9500	C25—C26	1.376 (3)
C6—C9	1.384 (3)	C26—H26	0.9500
C7—C9	1.430 (3)	C27—C28	1.485 (3)
C7—C10	1.432 (3)	C28—H28A	0.9800
C8—C10	1.391 (3)	C28—H28B	0.9800
C9—C13	1.508 (3)	C28—H28C	0.9800
C10—C14	1.498 (3)	C29—C30	1.483 (4)
C11—H11A	0.9800	C30—H30A	0.9800
C11—H11B	0.9800	C30—H30B	0.9800
C11—H11C	0.9800	C30—H30C	0.9800
C12—H12A	0.9800		
			
C6—O1—C11	118.99 (18)	C16—C15—H15	119.9
C8—O2—C12	118.56 (18)	C17—C15—H15	119.9
C27—O5—C18	118.60 (16)	C18—C16—C15	119.55 (19)
C29—O6—C25	117.98 (18)	C18—C16—H16	120.2
C3—C1—C4	122.6 (2)	C15—C16—H16	120.2
C3—C1—H1	118.7	C15—C17—C19	118.83 (18)
C4—C1—H1	118.7	C15—C17—C13	122.76 (17)
C5—C2—C4	122.0 (2)	C19—C17—C13	118.41 (17)
C5—C2—H2	119.0	C16—C18—C20	121.49 (18)
C4—C2—H2	119.0	C16—C18—O5	116.96 (18)
C1—C3—C6	118.6 (2)	C20—C18—O5	121.46 (18)
C1—C3—H3	120.7	C20—C19—C17	121.27 (19)
C6—C3—H3	120.7	C20—C19—H19	119.4
C1—C4—C2	121.4 (2)	C17—C19—H19	119.4
C1—C4—C7	119.1 (2)	C18—C20—C19	118.53 (19)
C2—C4—C7	119.5 (2)	C18—C20—H20	120.7
C2—C5—C8	119.3 (2)	C19—C20—H20	120.7
C2—C5—H5	120.3	C23—C21—C22	120.2 (2)
C8—C5—H5	120.3	C23—C21—H21	119.9
O1—C6—C9	115.61 (18)	C22—C21—H21	119.9
O1—C6—C3	122.9 (2)	C21—C22—C24	119.76 (18)
C9—C6—C3	121.4 (2)	C21—C22—C14	121.26 (18)
C4—C7—C9	118.11 (19)	C24—C22—C14	118.96 (17)
C4—C7—C10	117.59 (18)	C25—C23—C21	118.6 (2)
C9—C7—C10	124.28 (17)	C25—C23—H23	120.7
O2—C8—C10	116.90 (17)	C21—C23—H23	120.7
O2—C8—C5	121.51 (19)	C26—C24—C22	120.43 (19)
C10—C8—C5	121.3 (2)	C26—C24—H24	119.8
C6—C9—C7	119.92 (18)	C22—C24—H24	119.8
C6—C9—C13	117.15 (18)	C26—C25—C23	122.3 (2)
C7—C9—C13	122.02 (17)	C26—C25—O6	117.1 (2)
C8—C10—C7	119.91 (17)	C23—C25—O6	120.5 (2)
C8—C10—C14	117.62 (18)	C25—C26—C24	118.7 (2)
C7—C10—C14	121.36 (16)	C25—C26—H26	120.7
O1—C11—H11A	109.5	C24—C26—H26	120.7
O1—C11—H11B	109.5	O7—C27—O5	123.17 (19)
H11A—C11—H11B	109.5	O7—C27—C28	126.0 (2)
O1—C11—H11C	109.5	O5—C27—C28	110.85 (19)
H11A—C11—H11C	109.5	C27—C28—H28A	109.5
H11B—C11—H11C	109.5	C27—C28—H28B	109.5
O2—C12—H12A	109.5	H28A—C28—H28B	109.5
O2—C12—H12B	109.5	C27—C28—H28C	109.5
H12A—C12—H12B	109.5	H28A—C28—H28C	109.5
O2—C12—H12C	109.5	H28B—C28—H28C	109.5
H12A—C12—H12C	109.5	O8—C29—O6	122.6 (2)
H12B—C12—H12C	109.5	O8—C29—C30	127.1 (2)
O3—C13—C17	120.79 (17)	O6—C29—C30	110.2 (2)
O3—C13—C9	118.83 (17)	C29—C30—H30A	109.5
C17—C13—C9	120.35 (16)	C29—C30—H30B	109.5
O4—C14—C22	120.38 (17)	H30A—C30—H30B	109.5
O4—C14—C10	118.46 (17)	C29—C30—H30C	109.5
C22—C14—C10	121.13 (16)	H30A—C30—H30C	109.5
C16—C15—C17	120.28 (19)	H30B—C30—H30C	109.5
			
C4—C1—C3—C6	0.8 (3)	C7—C10—C14—O4	45.0 (3)
C3—C1—C4—C2	−175.9 (2)	C8—C10—C14—C22	55.0 (2)
C3—C1—C4—C7	3.5 (3)	C7—C10—C14—C22	−137.04 (18)
C5—C2—C4—C1	177.9 (2)	C17—C15—C16—C18	0.1 (3)
C5—C2—C4—C7	−1.4 (3)	C16—C15—C17—C19	1.7 (3)
C4—C2—C5—C8	3.2 (3)	C16—C15—C17—C13	−177.39 (19)
C11—O1—C6—C9	172.72 (18)	O3—C13—C17—C15	−171.31 (19)
C11—O1—C6—C3	−4.3 (3)	C9—C13—C17—C15	10.7 (3)
C1—C3—C6—O1	172.5 (2)	O3—C13—C17—C19	9.6 (3)
C1—C3—C6—C9	−4.4 (3)	C9—C13—C17—C19	−168.36 (17)
C1—C4—C7—C9	−4.1 (3)	C15—C16—C18—C20	−2.2 (3)
C2—C4—C7—C9	175.27 (18)	C15—C16—C18—O5	−178.72 (18)
C1—C4—C7—C10	177.48 (18)	C27—O5—C18—C16	−123.6 (2)
C2—C4—C7—C10	−3.2 (3)	C27—O5—C18—C20	59.9 (3)
C12—O2—C8—C10	−166.42 (19)	C15—C17—C19—C20	−1.5 (3)
C12—O2—C8—C5	19.1 (3)	C13—C17—C19—C20	177.64 (18)
C2—C5—C8—O2	174.0 (2)	C16—C18—C20—C19	2.4 (3)
C2—C5—C8—C10	−0.2 (3)	O5—C18—C20—C19	178.76 (17)
O1—C6—C9—C7	−173.40 (17)	C17—C19—C20—C18	−0.5 (3)
C3—C6—C9—C7	3.7 (3)	C23—C21—C22—C24	−0.9 (3)
O1—C6—C9—C13	−4.1 (3)	C23—C21—C22—C14	177.33 (17)
C3—C6—C9—C13	173.02 (18)	O4—C14—C22—C21	−151.77 (18)
C4—C7—C9—C6	0.6 (3)	C10—C14—C22—C21	30.3 (3)
C10—C7—C9—C6	178.93 (18)	O4—C14—C22—C24	26.5 (3)
C4—C7—C9—C13	−168.20 (17)	C10—C14—C22—C24	−151.44 (17)
C10—C7—C9—C13	10.1 (3)	C22—C21—C23—C25	1.6 (3)
O2—C8—C10—C7	−178.93 (17)	C21—C22—C24—C26	−0.5 (3)
C5—C8—C10—C7	−4.4 (3)	C14—C22—C24—C26	−178.78 (17)
O2—C8—C10—C14	−10.8 (3)	C21—C23—C25—C26	−0.9 (3)
C5—C8—C10—C14	163.68 (19)	C21—C23—C25—O6	−177.03 (16)
C4—C7—C10—C8	6.0 (3)	C29—O6—C25—C26	119.9 (2)
C9—C7—C10—C8	−172.35 (18)	C29—O6—C25—C23	−63.8 (3)
C4—C7—C10—C14	−161.67 (17)	C23—C25—C26—C24	−0.5 (3)
C9—C7—C10—C14	20.0 (3)	O6—C25—C26—C24	175.78 (17)
C6—C9—C13—O3	−113.6 (2)	C22—C24—C26—C25	1.2 (3)
C7—C9—C13—O3	55.5 (3)	C18—O5—C27—O7	−0.9 (3)
C6—C9—C13—C17	64.4 (2)	C18—O5—C27—C28	178.60 (18)
C7—C9—C13—C17	−126.51 (19)	C25—O6—C29—O8	2.4 (3)
C8—C10—C14—O4	−122.9 (2)	C25—O6—C29—C30	−175.0 (2)

### Hydrogen-bond geometry (Å, º)

**Table:**

*D*—H···*A*	*D*—H	H···*A*	*D*···*A*	*D*—H···*A*
C11—H11*C*···O4^i^	0.98	2.36	3.320 (3)	166
C12—H12*A*···O3^ii^	0.98	2.53	3.380 (3)	145
C3—H3···O7^iii^	0.95	2.47	3.369 (3)	158
C21—H21···O8^iv^	0.95	2.53	3.364 (3)	146

Symmetry codes: (i) *x*, *y*−1, *z*; (ii) *x*, −*y*, *z*−1/2; (iii) *x*, −*y*−1, *z*−1/2; (iv) −*x*+1, −*y*, −*z*+1.
